# Molecular Basis of Olfactory Chemoreception in the Common Bed Bug, *Cimex lectularius*

**DOI:** 10.1038/srep45531

**Published:** 2017-04-06

**Authors:** Feng Liu, Zhou Chen, Nannan Liu

**Affiliations:** 1 Department of Entomology and Plant Pathology, Auburn University, Auburn, AL 36849, USA

## Abstract

As one of the most notorious ectoparasites, bed bugs rely heavily on human or animal blood sources for survival, mating and reproduction. Chemoreception, mediated by the odorant receptors on the membrane of olfactory sensory neurons, plays a vital role in their host seeking and risk aversion processes. We investigated the responses of odorant receptors to a large spectrum of semiochemicals, including human odorants and plant-released volatiles and found that strong responses were sparse; aldehydes/ketones were the most efficient stimuli, while carboxylic acids and aliphatics/aromatics were comparatively less effective in eliciting responses from bed bug odorant receptors. In bed bugs, both the odorant identity and concentrations play important roles in determining the strength of these responses. The odor space constructed based on the responses from all the odorant receptors tested revealed that odorants within the same chemical group are widely dispersed while odorants from different groups are intermingled, suggesting the complexity of odorant encoding in the bed bug odorant receptors. This study provides a comprehensive picture of the olfactory coding mechanisms of bed bugs that will ultimately contribute to the design and development of novel olfactory-based strategies to reduce both the biting nuisance and disease transmission from bed bugs.

Chemoreception is critical for insects as it plays a vital role in locating hosts, finding mates, identifying oviposition sites and avoiding natural enemies. The common bed bug, *Cimex lectularius*, as a resurgent parasite in both human beings and animals, can detect a large panel of stimuli from human odorants as well as the plant-released volatiles used as chemical repellents for mosquitoes or other hematophagous arthropods^1^,^2^,^3^,^4^. The olfactory receptor neurons (ORNs) housed in the olfactory sensilla on the bed bug’s antennae are extremely sensitive to several chemical classes, including the aldehydes/ketones and amines in human odors and several terpene-derived stimuli extracted from plants^3^.

The olfactory receptors on the olfactory receptor neuron membrane are responsible for detecting chemical stimuli in the insect’s surroundings. Odorant receptors (ORs), as the most extensively investigated clade of olfactory genes, are known to play a fundamental role in the chemoreception of a number of insect species, including the common bed bug. Odorants with biological meanings for insects are specifically recognized by the ORs in the neuron membrane and trigger the firing process in the ORNs^5^,^6^,^7^, providing the primary olfactory information for further odor identification in the central nervous system (CNS).

Previous studies on olfactory system have indicated that bed bugs possess a degenerative olfactory system with far fewer olfactory sensilla and ORs than many other insect species^8^,^9^. Indeed, there have been reported to be only 44 sensilla (29 E sensilla, 6 D sensilla and 9 C sensilla) on the second flagellum^8^ and just 10 olfactory sensilla (2 E sensilla, 2 D sensilla and 6 C sensillum) on the pedicel of bed bug antennae^10^. A transcriptome analysis for the bed bug antennae identified 16 ORs with significant expression^11^ and a recently published bed bug genome sequence revealed there to be around 47 bed bug ORs, the number of which was substantially reduced compared to that of phytophagous hemipterans, such as pea aphid^9^. As an obligate blood-feeding insect, the intermediate number of bed bug chemoreceptors had been suggested to be in line with its moderate complexity of chemical ecology^9^, greatly contrasting with some other insects with very complex chemical environment, like the yellow fever mosquitoes (*Aedes aegypti*) possessing 131 ORs and honeybees (*Apis mellifera*) owning 170 ORs^12^,^13^. Even though the olfactory neuronal responses of bed bugs to human odorants or some chemical insect repellents have been extensively characterized, very little research has sought to decipher the molecular basis of chemoreception in the common bed bug. Previous work by our group investigated the function of two bed bug ORs (OR5 and OR9b, previously named as OR1 and OR2, respectively) in response to 42 human odorants, which revealed only very limited information regarding the molecular basis of chemoreception^3^. To gain a better understanding of the function of bed bug ORs and the molecular basis of chemoreception in the common bed bug, in this study we successfully characterized the function of 15 bed bug ORs in response to a much larger chemical panel consisting of 148 odorants from both human odorants and botanical chemical stimuli, providing a much more informative and general picture of the sensory ecology of bed bugs.

## Results

### Evolutionary stability of bed bug OR family

**Figure 1:**
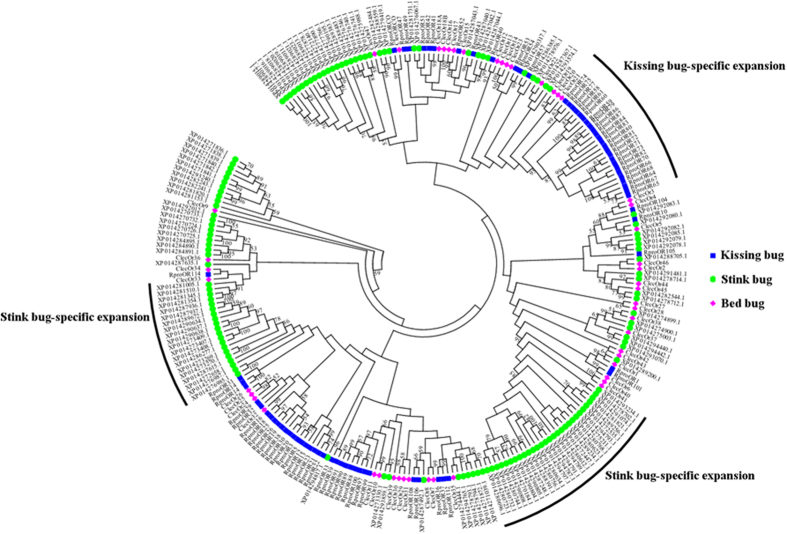
Phylogenetic analyses of the odorant receptor genes of bed bugs, kissing bugs and stink bugs. All 47 bed bug ORs (shown in pink) were retrieved from the bed bug genome annotation (www.hasc.org); the 76 ORs from the kissing bug (*Rhodnius proxilus*, shown in blue) were retrieved from Vectorbase (www.vectorbase.org); and the 133 ORs from the stink bug (*Halyomorpha halys*, shown in green) were retrieved from the NCBI website (http://www.ncbi.nlm.nih.gov). The tree was constructed with MEGA6 based on a ClustalW alignment of the amino acid sequences. Numbers above branches represent the percentage of 1,000 bootstrap replication trees in that branch, with only those above 50% shown.

Based on the existing genomic data, we performed phylogenetic analyses of the ORs of two hematophagous Hemipterans, the common bed bug (*C. lectularius*) and the kissing bug (*Rhodnius proxilus*), and one phytophagous Hemipteran, the stink bug (*Halyomorpha halys*). The 47 bed bug ORs were used to build a phylogenetic tree with 72 ORs from the kissing bug (www.vectorbase.org) and 133 ORs from the stink bug (http://www.ncbi.nlm.nih.gov/). According to this phylogenetic tree, odorant receptor co-receptor (ORCO) genes from all three organisms are clustered together due to their highly conserved amino acid sequence (Fig. 1). Specific OR gene expansion was observed in both kissing bugs and stink bugs, with at least two branches of ORs specifically evolved in stink bugs, which may be relevant to their phytophagy comparing to bed bugs and kissing bugs, and one branch of kissing bug ORs showed no close relatives from bed bugs or stink bugs. However, we found that no bed bug-specific OR gene expansion was demonstrated in the phylogenetic tree. Most of the bed bug ORs were clearly clustered with specific ORs from either kissing bugs or stink bugs, which suggests a slow rate of evolution in the bed bug OR gene family. The relatively conservative nature of OR gene family also suggests a comparatively stable chemosensory ecology in bed bugs, which may result from their obligate blood-feeding requirement, narrow host spectrum and relatively simple habitat environment (always close to their hosts).

### Sensory spectrum of bed bug ORs to odorant stimuli

The *Xenopus* oocyte expression system has been successfully used to characterize the function of ORs from multiple insect species^14^,^15^,^16^. In our study, a total of 47 ORs were tested, 21 of which showed successful cDNA amplification and correct amino acid sequences. Among these 21 ORs, 15 of them produced specific odorant-induced response profiles when co-expressed with ORCO in *Xenopus* oocytes. The remaining six ORs gave no responses to any odor panel component. Overall, 3108 odorant-receptor combinations were individually tested in the two-electrode voltage-clamp system, of which 2220 functional interactions displayed significant variation in the absolute amplitude of the OR current responses.

**Figure 2:**
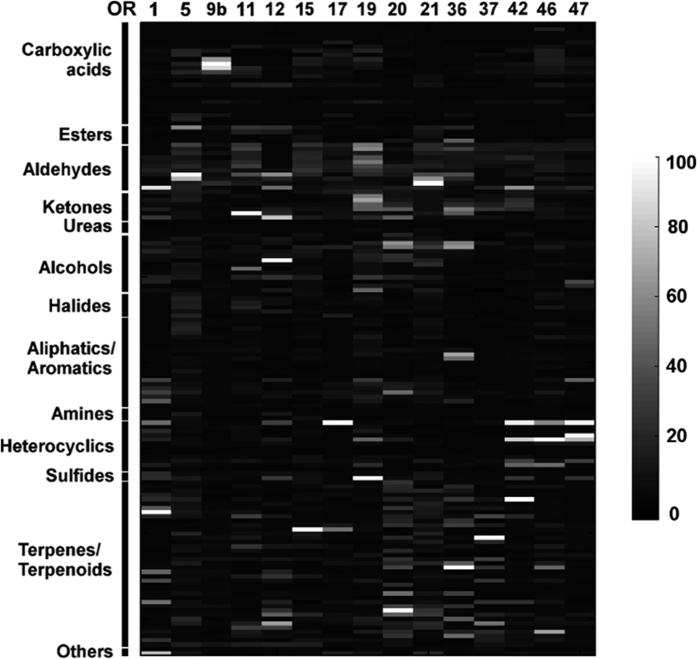
A heatmap presentation of the normalized response profiles of bed bug ORs to odorants. Response intensity is color-coded according to the continuous color scale on the right, and represents the mean activity measured by a two-electrode voltage clamp. Receptors, odorants and numerical values are provided in Supplementary Table 2. n = 3–6; odorants that elicit responses ≥100 nA, n = 6. All odorants were tested at a 1:10,000 v/v dilution. The solvent, 0.1% DMSO Ringer’s solution, produced no stimulation in any of the ORs.

**Figure 3:**
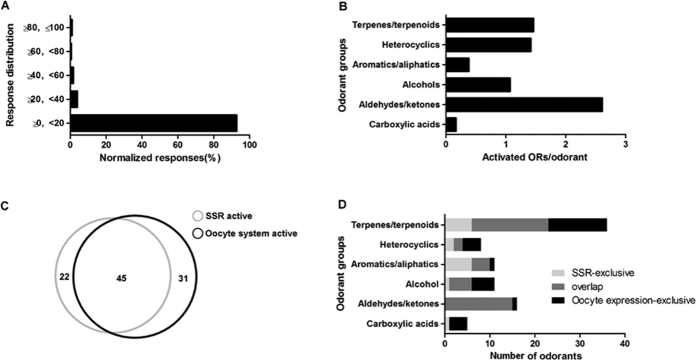
Summary of the current responses of bed bug ORs to odorant stimuli. (**A**) Distribution of current responses with different strengths evoked by various odorant/OR combinations. Strong responses (≥20% RU) were sparse among all the odorant-OR combinations. (**B**) Effectiveness of odorants in different chemical groups in eliciting responses ≥20% RU. The average number of ORs activated by individual odorants was calculated by dividing the total number of strong responses by the total number of odorants within the chemical group. For instance, all 17 aldehydes/ketones elicited 44 strong responses (≥20% RU), so the average number of ORs activated by aldehydes/ketones would be 2.6, as shown in the bar chart. (**C**) Overlap of the SSR-active odorants and the oocyte expression system active odorants. Odorants that were active in both SSR and oocyte expression system are in the overlapping area of the cycles. Areas with no overlaps represent SSR-exclusive (gray cycle) or oocyte expression system (black cycle) exclusive odorants. (**D**) Odorants within major chemical groups of odorants that are active in SSR or oocyte expression system. Light gray bars indicate odorants that are only active in the SSR system and black bars signify odorants that are only active in the oocyte expression system. Dark gray bars are odorants that are active in both systems.

To facilitate comparisons between all the OR-odorant pairs, responses were normalized by defining the maximal odorant response for each receptor as 100 response units (RU). On this basis, strong current responses were relatively sparse (Fig. 2), with only 3.96% of the OR-odorant pairs displaying responses between 20% and 40% of the maximal responses; 1.71% between 40% and 60%; 0.59% between 60% and 80%; and only 0.95% above 80% (Fig. 3A).

This normalization allowed us to assess bed bug OR responses among different chemical groups. We found the average frequency of strong responses (>20 RU) evoked by odorants in different chemical groups varied considerably. Aldehydes/ketones were the most efficient group, eliciting strong responses (>20 RU) from an average of 2.6 ORs per odorant, and alcohols, terpenes/terpenoids and heterocyclics also triggered strong responses (>20 RU) on at least 1 OR per odorant, with aliphatics/aromatics (0.38 OR/odorant) and carboxylic acid (0.17 OR/odorant) falling well behind (Fig. 3B).

Two previous studies using nearly the same panel of odorants to test the neuronal responses of bed bugs via single sensillum recording (SSR)^2^,^3^, enabled us to directly compare the sensory spectra of ORNs and ORs. Unsurprisingly, most of the odorants (45 out of 67) eliciting active ORNs responses (≥50 spikes/s or 20% of the maximal responses) were also very effective in activating the ORs (Fig. 3C). A closer examination of the major odorant groups tested in both experiments revealed some interesting variations in the receptive spectra within these odorant groups (Fig. 3D). For example, all the aldehydes, most of the terpenes/terpenoids and alcohols that were active in ORNs (SSR system) were also effective in ORs (oocyte expression system) but only a small part of aromatics/aliphatics that were active in ORNs successfully activated the ORs. Considering that the response spectra of only 15 ORs (about one third of the total ORs) were characterized in this study, it is very likely that most of the odorants active in the SSR system will be ultimately covered by the oocyte expression system. This may not be the whole story, however, as certain odorants that are active in ORs were not perceived by the ORNs, possibly due to a major disadvantage of the oocyte expression system in that only naked ORs are tested with no involvement of other factors, such as odorant binding proteins, that could also play a selective role in delivering the odorants to ORs on the neuron membrane. It thus seems reasonable to infer that ORs possess a larger response spectrum than ORNs.

### Tuning breadth of bed bug OR repertoire

**Figure 4:**
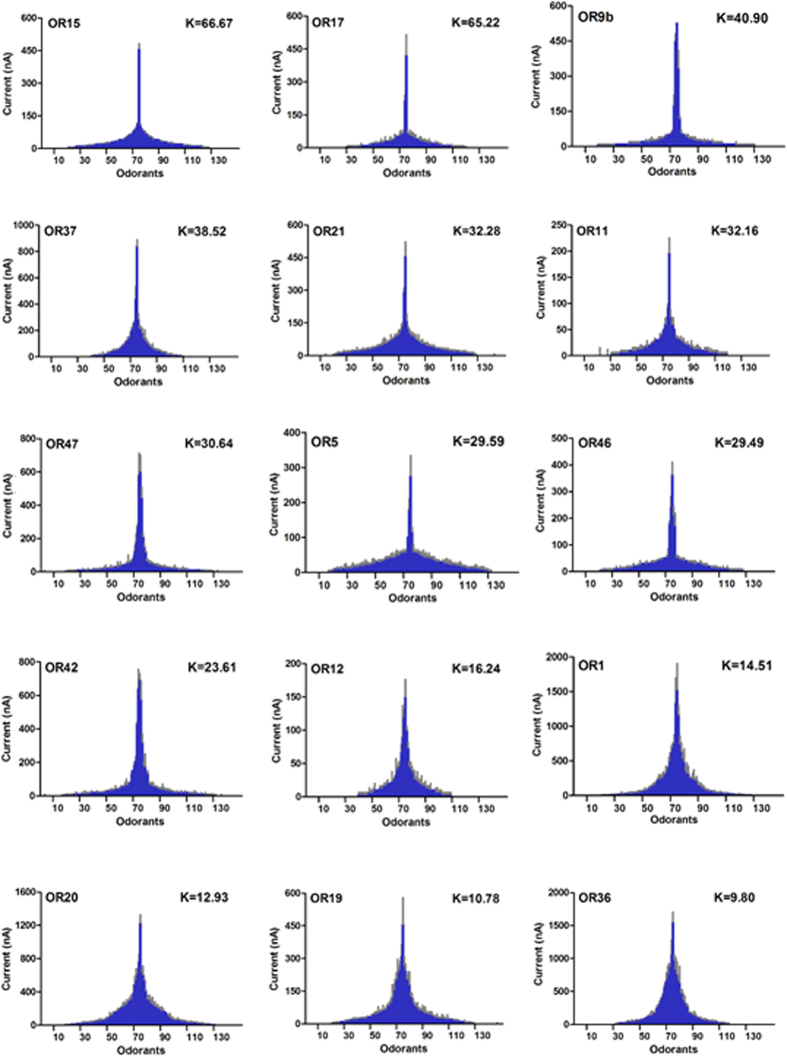
Tuning curves of bed bug ORs. Non-normalized OR responses are presented as in Carey *et al*.^18^ and Wang *et al*.^15^. The 148 odorants are displayed along the x axis, with those eliciting the strongest response are near the center and those with weaker responses near the edges; note that the order of odorants is different for each receptor. The kurtosis value, k, a statistical measure of ‘peakedness’ is shown alongside each OR. The tuning curve of each OR is arranged from small to large kurtosis value.

In order to compare the specific response spectra of individual bed bug ORs to the odorants, OR tuning curves were generated (Fig. 4) following the procedure described by Wang *et al*.^15^. The results indicated that several ORs (e.g. OR15, 17, 9b, 37) were quite specialized, with each OR responding strongly to only a very few odorants. For example, OR15 displayed a particular sensitivity to β-caryophyllene, OR17 responded strongly coumarin and OR37 was very sensitive to citral (Fig. 4, Table S2). At the other end of the spectrum, OR1, OR19, OR20, and OR36 were more likely to be classified as generalists as they showed responses to multiple odorants across several chemical groups. For example, OR36 was found to respond strongly to about 30 structurally diverse odorants, including aldehydes, ketones, aliphatics/aromatics, terpenes/terpenoids, and alcohols (Fig. 4, Table S2). All the tuning curves for the 15 bed bug ORs clearly demonstrated that the receptive range of bed bug ORs follows a continuing pattern and varies smoothly from very narrowly to broadly tuned, which is consistent with previous findings in both the fruit fly (*Drosophila melanogaster*)^17^ and the malaria mosquito (*An. gambia*)^15^,^18^.

Interestingly but perhaps not surprisingly, some narrowly tuned ORs showed extremely strong responses to compounds that are biologically important for bed bugs. For example, both OR9b and OR21 responded strongly to decanal (Fig. 4, Table S2), which is a very important component of airborne bed bug aggregation pheromone that has been linked to bed bugs’ aggregation behavior^19^, and OR37 is narrowly tuned to citral (Fig. 4, Table S2), which exhibits very strong repellency for bed bugs (unpublished data).

### Odor coding and odorant identity

When we examined the response profiles of bed bug ORs to different odorants, it is evident that odorant identity has a considerable impact on the responses of individual OR, especially among some structurally similar odorants. For instance, OR15 was exclusively sensitive to β-caryophyllene but showed only a very weak response to (−)-caryophyllene oxide (Table S2). Another significant example is OR36, which presented a remarkable response to trans-3-octene and trans-4-octene but a very weak response to trans-2-octene, which suggests that the double bond position in the molecule controls the activation efficiency for this OR (Table S2).

The bed bug ORs not only exhibited strict requirements for the chemical structures, but also for the stereotypes of isomers of the same chemical. For example, (+)-menthone evoked a remarkable current response (242 nA) from OR46 while (−)-menthone produced only a minor current response (25 nA) (Table S2). Similarly, (+)-β-pinene (315 nA) elicited a much stronger response from OR20 than (−)-β-pinene (55 nA) achieved (Table S2). These results further suggested the superior capacity of bed bug ORs to discriminate between odorants with subtle variances in their chemical structure.

**Figure 5:**
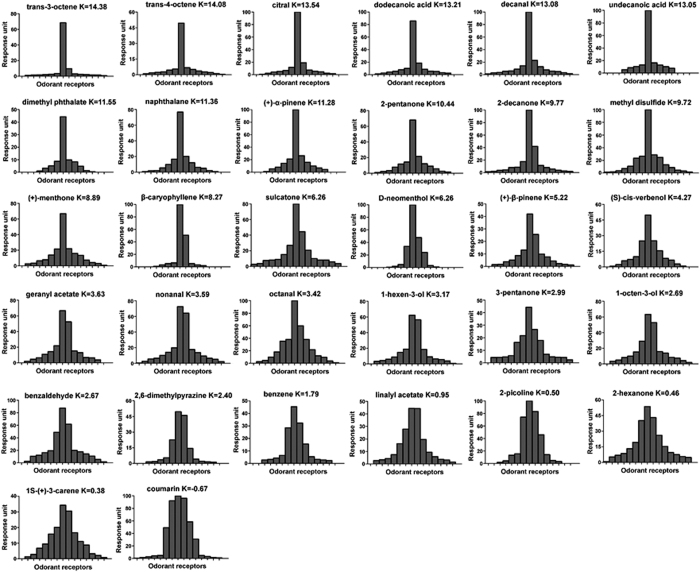
Tuning curves of odorants. The normalized responses of the 15 ORs are ordered along the x-axis according to the magnitude of the response generated for each odorant. The receptor with the strongest response is at the center of the distribution and those with the weakest at the edges; note that the order of receptors is different for each odorant. The kurtosis value is indicated in each graph. 32 odorants with tuning curves ranging from very narrow to very broad are selectively presented, with the tuning curve of each arranged from small to large kurtosis value.

Given that different odorants are recognized or encoded by different ORs in the common bed bug, a comparison of the receptor spectrum response to different odorants could produce some interesting results. We therefore generated a set of “odorant tuning curves” to represent the odorant-activated receptors with differential responses, which is the reciprocal of receptor tuning curves and considered as a complementary analysis approach in identifying receptors and odorants that are important for innate insect behavioral responses^18^. Tuning curves of 32 odorants that had been shown to be particularly effective in activating single or multiple ORs were selectively presented according to their tuning breadth (Fig. 5). As with the receptor tuning curve, some odorants were found to be narrowly recognized by only a very few ORs while others were broadly recognized by multiple ORs. For instance, trans-3-octene and trans-4-octene were only recognized by OR36 and citral was solely encoded by OR37. Both trans-3-octene and trans-4-octene elicited strong neuronal responses in SSR^3^; both are isomers of those known components of human emanation (3-octene and 4-octene)^20^, which may hint at their possible role in the host location behavior of bed bugs. Interestingly, citral, as a very efficient repellent for bed bugs, was found to be a “narrowly tuned” odorant recognized by a “narrowed tuned” receptor, OR37.

### Dose-dependent response of ORs to odorant stimuli

**Figure 6:**
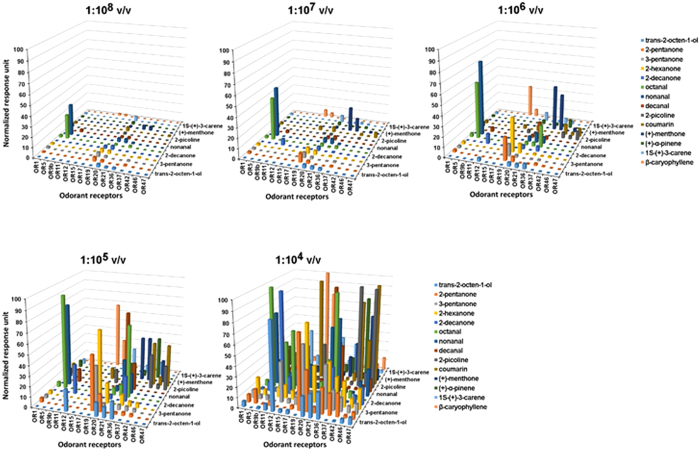
Dose-dependent activity of odorant receptors. Normalized responses of 15 ORs to a subset of 14 odorants displaying dose-dependent characteristics. Odorants are listed on the Z-axis sequentially from trans-2-octen-1-ol to β-caryophyllene (n = 3–6).

**Figure 7:**
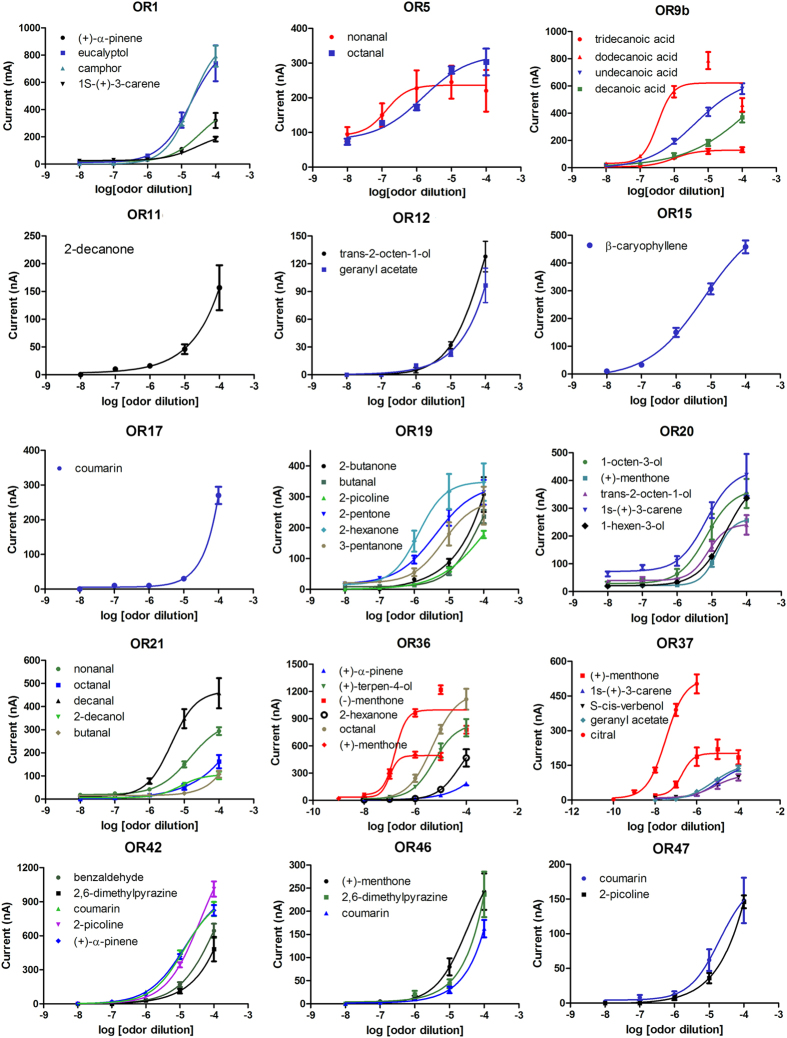
Dose-dependent responses. Dose-response curves of ORs to different odorants (means ± SEM, n = 3–6). EC_50_ values are expressed as dilutions of pure reagents in Ringer’s solution^15^. Red dose–response curves indicate ligands whose EC_50_ values are in the range of 10^−7^ dilution or below. OR1: (+)-α-pinene, EC_50_ = 3.41 × 10^−5^; eucalyptol, EC_50_ = 1.88 × 10^−5^; camphor, EC_50_ = 1.94 × 10^−5^; 1s-(+)-3-carene, EC_50_ = 2.93 × 10^−5^; OR5: nonanal, EC_50_ = 1.32 × 10^−7^; decanal, EC_50_ = 1.53 × 10^−6^; OR12: trans-2-octen-1-ol, EC_50_ = 1.03 × 10^−4^; OR15: β-caryophyllene, EC_50_ = 6.46 × 10^−6^; OR19: butanal, EC_50_ = 6.56 × 10^−5^; 2-picoline, EC_50_ = 1.11 × 10^−4^; 2-pentanone, EC_50_ = 4.13 × 10^−6^; 2-hexanone, EC_50_ = 1.33 × 10^−6^; 3-pentanone, EC_50_ = 6.84 × 10^−6^; OR20: 1-octen-3-ol, EC_50_ = 6.95 × 10^−6^; (+)-menthone, EC_50_ = 1.44 × 10^−5^; trans-2-octen-1-ol, EC_50_ = 7.65 × 10^−6^; 1s-(+)-3-carene, EC_50_ = 6.85 × 10^−6^; 1-hexen-3-ol, EC_50_ = 2.61 × 10^−5^; OR21: nonanal, EC_50_ = 1.48 × 10^−5^; decanal, EC_50_ = 4.27 × 10^−6^; 2-decanol, EC_50_ = 7.55 × 10^−6^; OR36: octanal, EC_50_ = 4.79 × 10^−6^; (−)-menthone, EC_50_ = 1.64 × 10^−7^; (+)-terpen-4-ol, EC_50_ = 5.49 × 10^−6^; 2-hexanone, EC_50_ = 5.12 × 10^−5^; (+)-menthone, EC_50_ = 9.92 × 10^−8^; OR37: citral, EC_50_ = 3.32 × 10^−8^; (+)-menthone, EC_50_ = 1.93 × 10^−7^; 1s-(+)-3-carene, EC_50_ = 1.13 × 10^−5^; S-cis-verbenol, EC_50_ = 7.10 × 10^−6^; geranyl acetate, EC_50_ = 6.01 × 10^−6^; OR42: coumarin, EC_50_ = 1.45 × 10^−5^; 2-picoline, EC_50_ = 3.89 × 10^−5^; (+)-α-pinene, EC_50_ = 1.32 × 10^−5^; OR46: (+)-menthone, EC_50_ = 3.46 × 10^−5^; OR47: coumarin, EC_50_ = 2 × 10^−5^.

Numerous studies have indicated that concentration is a critical factor in determining the responses of ORs to odorants^15^,^16^,^18^. Our results confirmed this: the responses of ORs were dramatically influenced by the odorant concentrations, with low concentrations eliciting very weak responses from ORs while high doses (1:10^3^ or 1:10^4^ v/v) activated a large number of ORs (Fig. 6). To further compare the sensitivity of ORs to the odorant stimuli, the EC_50_ value of odorants for different ORs were calculated (Fig. 7). The dose-response curves of ORs to different odorants revealed that certain ORs only responded to certain odorants at high doses. For example, OR17 and OR11 only displayed strong responses to coumarin and 2-decanone at a dose of 1:10^4^ v/v. However, other ORs appeared to be extremely sensitive to odorants with a low dose: OR36 was activated by (+)-menthone and (−)-menthone with EC_50_ values of 9.67 × 10^−8^ and 1.64 × 10^−7^ v/v, respectively, and OR37 was activated by citral and (+)-menthone with EC_50_ values of 3.32 × 10^−8^ and 1.93 × 10^−7^ v/v, respectively (Fig. 7, Fig. S1). As all these EC_50_ values are in the nanomolar range, they are likely to be the cognate ligands for these ORs^21^.

### Odor space of bed bugs

As indicated in this study, odorants are usually recognized combinatorically by multiple ORs, which is consistent with the scenario found in *Drosophila*, mosquito and mammalian ORs^15^,^17^,^18^,^22^. To examine the relationship between the chemical nature of odorant stimuli and OR responses, we constructed a multidimensional odor space to display the non-normalized current responses of the 15 functional ORs identified in the *Xenopus* oocyte expression system, mapping the Euclidean distances (in nanoamperes, nA) between all responsive pairs of ORs and odorants.

**Figure 8:**
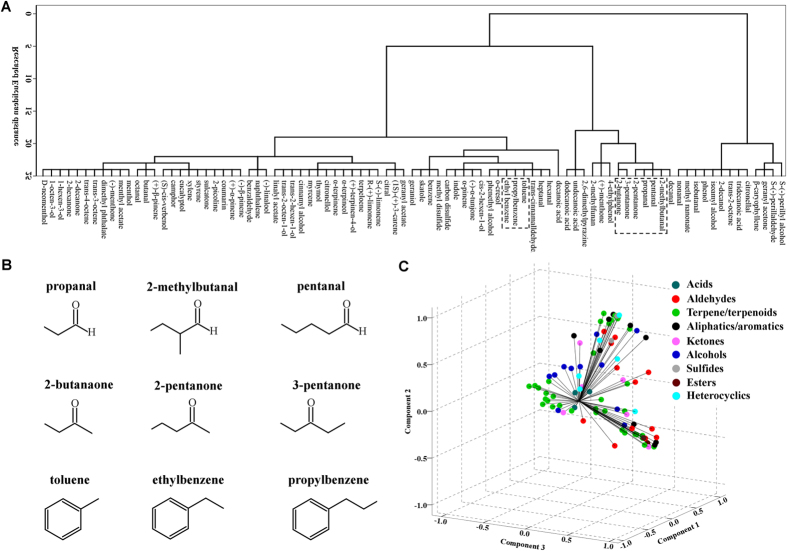
Bed bug odor space. (**A**) Hierarchical cluster analysis for odorants based on the Euclidean distance between 87 odors, which elicited current response of ≥100 nA on at least one OR. (**B**) Represented odorants with similar chemical structure are clustered together in the Hierarchical cluster analysis. (**C**) Relationships among odorants of indicated chemical classes at a dose of 1:10^4^ v/v, revealed by PCA. In PCA, vectors quantifying the responses of the 15 odorant receptors to each odor in (**A**) are projected onto a three-dimensional space as described in ref. 15. This three-dimensional representation captures 69.5% of the variance in the original 15-dimensional data set.

Although the odor space built in this study represents only a subset of all possible OR-odorant response combinations, and thus comprises only part of the bed bug’s overall chemosensory input, it is reasonable to assume that odorants clustered together with small Euclidean distances within this odor space generally share significant chemical characteristics and are thus difficult for bed bugs to differentiate. To visualize the relationships among odorants in this space, a hierarchical cluster analysis was performed on the odorants based on the Euclidean distance within each odorant pair (Fig. 8A). We found that odorants in the same chemical group often, though not always, clustered together. Moreover, an inspection of these clusters revealed many examples of structurally similar molecules that are tightly clustered, such as several of the aliphatic aldehydes and ketones (propanal, 2-methylbutanal, pentanal, 2-butanone, 2-pentanone, 3-pentanone) and aromatics (toluene, ethylbenzene, propylbenzene) groups with only minor variations in their side carbon chain (Fig. 8B).

As another way of analyzing the relationships among odorants, principle component analysis (PCA) was applied to represent the 15-dimensional odor space in a three-dimensional odor space that captured about 69.5% of the original variances (Fig. 8C). In this 3D odor space, odorants from each chemical group were more likely to disperse across the whole odor space, even though limited clustering was observed for a small number of odorants. Interestingly, in some cases odorants from several different chemical groups appeared to intermingle closely, with no significant separation, suggesting that although chemical class is a critical factor, it is not the only factor involved in the odorant encoding process of bed bug odorant receptors.

## Discussion

Semiochemicals play a critical role in the host seeking and risk aversion process of insects. For bed bugs, human odorants were served as important cues for host seeking while chemical repellents, like compounds from alarm pheromone, delivered dangerous information for potential risks. In previous work^2^,^3^, we extensively described the olfactory neuronal response of bed bugs to human odorants and several potential chemical repellents. However, as yet there have been few investigations that focused specifically on the molecular basis of bed bugs’ chemoreception, with only preliminary studies reported^3^,^11^. Therefore, this study provides the first overview of the molecular basis of bed bugs’ chemoreception by investigating the responses of 15 odorant receptors to a large panel of odorant stimuli from both human emanations and plant volatiles.

When comparing the olfactory neuronal responses of olfactory sensilla with the current responses of ORs to the same panel of odorant stimuli tested in this study, it is clear that around 67% of the odorants elicited strong neuronal responses likely to evoke strong current responses by the odorant receptor, supporting that ORs are indeed the very important target of these odorants on the neuronal membrane and activated ORs are responsible for the corresponding neuronal firing. As more bed bug ORs are going to be functionally characterized, we posit that certain ORs will be identified for most of these odorants that are effective in triggering neuron firing. However, we also found a number of odorants that appeared to have no effect on the ORNs but activated ORs in this study. For example, dimethyl phthalate at a dose of 1:10^4^ v/v evoked a very strong current response (552 nA) on OR36, but none of the olfactory sensilla exhibited a very strong response to dimethyl phthalate in the single sensillum recording^3^. A similar phenomenon was also observed in coumarin, which was found to be a very effective stimulus for OR17, OR42, and OR47 but showed very weak or no neuronal responses from the olfactory sensillum^3^. There are several possible reasons for this inconsistency: (1) bed bugs seldom encounter chemicals like dimethyl phthalate or coumarin at such high doses in their natural habitat; (2) there are no specific odorant binding proteins (OBPs) in the sensillum lymph that are responsible for transporting these odorants to their target ORs; (3) ORNs in bed bug sensilla may derive their ligand specificity from the expression of multiple ORs per neuron as found in *D. melanogaster*^23^,^24^, which may render odorants potent in activating olfactory sensillum but not individual OR. Future studies on the actual doses of odorants in the insect’s natural environment and a more detailed characterization of the function of bed bugs’ OBPs would help address this question.

In this study, we further demonstrated that aldehydes/ketones are likely the most important stimuli released from human bodies that bed bugs are sensitive to, which is very consistent with our earlier finding from the ORNs^3^. Moreover, certain odorants from the aldehydes/ketones (such as nonanal, sulcatone), some alcohols (such as 1-octane-3-ol) and heterocyclics (such as skatole) that bed bug ORs were very sensitive to have also been reported to be active attractants for mosquitoes^25^,^26^,^27^,^28^. It therefore seems likely that odorants from aldehydes/ketones, alcohols and heterocyclics are very important in the host locating process of bed bugs. A finely designed behavior bioassay is needed to further test this hypothesis.

In addition, terpenes and terpenoids were found to be very active in evoking current responses from the bed bug ORs, confirming their high potency in triggering firing in the ORNs housed in the olfactory sensilla on bed bug antennae^2^. Previous behavioral studies on both bed bugs and mosquitoes have indicated that plant-released terpenes or terpenoids stimuli are very repulsive^29^,^30^,^31^ (unpublished bioassay data for bed bugs). Several terpenes or terpenoids (including, for example, citral, (+)-menthone, geranyl acetate and 1s-(+)-3-carene) have displayed a higher efficiency than DEET, one of the most important and successful “all round” synthetic chemical repellents^32^, in repelling bed bugs in a two-choice behavior bioassay (unpublished bioassay data for bed bugs). Given that certain bed bug ORs are specifically sensitive to terpenes or terpenoids that initiate firing in the ORNs, this may be responsible for the aversive behaviors displayed. This suggests that terpenes or terpenoids are very promising candidates for screening new chemical repellents for bed bug control.

As indicated in this study and in previous studies of fruit flies and mosquitoes^15^,^17^, the concentrations of odorants are critical in triggering responses from odorant receptors. Some ORs can be activated only at high doses, while others are capable of respond to exceptionally low doses of odorants. Considering the actual doses of odorants in the natural environment are generally very low, possibly even lower than the already low doses used in this experiment, we believe that the responses elicited by low doses of odorants are, to some extent, closer to the naturally occurring levels of odorant reception in the bed bugs’ ORNs.

Although the multi-dimensional odor space generated based on the relationships of odorants and OR responses for this study have provided remarkable information about the ability of bed bugs to discriminate these odorants, it is important to note that the odor space we defined here covered only part of the bed bug’s olfactory range. As more bed bug ORs are functionally characterized, we expect to build a much more complete picture of how these odorants are encoded by the ORs. Once we have clarified the reception spectrum of all these bed bug ORs, we can then move on to determine how bed bugs respond to: 1) plumes (blends) of odorants rather than single odorants used in isolation; and 2) rapidly changing concentrations of odorants in their natural environment rather than the manually defined concentrations utilized in the *Xenopus* oocyte *ex vivo* expression system used in this study. To address these questions, more sophisticated approaches such as patch clamp recording directly in the antennal lobe or calcium imaging will be helpful.

## Materials and Methods

### Insects

The *C. lectularius* colony utilized in this study originally came from Ft. Dix, New Jersey, USA. It is susceptible to pyrethroid insecticides^33^. The bed bugs were fed with rabbit blood once every week in the laboratory. Blood was purchased from Hema Resource and Supply Company (Aurora, OR). All the common bed bugs were reared at 25 ± 2 °C under a photoperiod of 12:12 (L: D).

### RT-PCR, cDNA cloning and cRNA synthesis

Adult bed bugs were cold anesthetized with ice. Olfactory tissues (antennae) were hand dissected and stored in dry ice for RNA extraction. Total RNA was extracted from the adult olfactory appendages using the acidic guanidine thiocyanate-phenolchloroform method^34^ and used for oligo (dT)-primed cDNA synthesis with SuperScript III reverse transcriptase (Invitrogen) to generate templates for subsequent PCR reactions using full-length primers with a specific restriction enzyme cutting site added (Table S1). The purified PCR products were cloned into pT7Ts vector (a generous gift from Dr. Wang at the Institute of Plant Protection, CAAS, China), with a Kozak sequence added behind the cutting site in the forward primer. The recombinant plasmids were sequenced (the Genomic Facility of Auburn University) and all OR sequences were confirmed through Vectorbase before constructing linearized plasmids for synthesizing cRNAs using mMESSAGE mMACHINE T7 kit as instructed by the manufacture (Ambion, Carlsbad, CA).

### *Xenopus* oocyte expression system and two-electrode voltage-clamp

Mature healthy oocytes (stage V–VII) (Nasco, Salida, CA) were treated with collagenase I (GIBCO, Carlsbad, CA) in washing buffer (96 mM NaCl, 2 mM KCl, 5 mM MgCl_2_, and 5 mM HEPES [pH = 7.6]) for about 1 h at room temperature. After being cultured overnight at 18 °C, the oocytes were microinjected with 10 ng cRNAs of both ORs and ORCO. After injection, the oocytes were incubated for 4–7 days at 18 °C in 1X Ringer’s solution (96 mM NaCl, 2 mM KCl, 5 mM MgCl_2_, 0.8 mM CaCl_2_, and 5 mM HEPES [pH = 7.6]) supplemented with 5% dialyzed horse serum, 50 mg/ml tetracycline, 100 mg/ml streptomycin and 550 mg/ml sodium pyruvate. Whole-cell currents were recorded from the injected *Xenopus* oocytes with a two-electrode voltage clamp. Odorant-induced currents were recorded with an OC-725C oocyte clamp (Warner Instruments, Hamden, CT) at a holding potential of −80 mV. Odorants were dissolved in DMSO at a 1:10 ratio to make stock solutions and then the stock solution was further diluted with 1× Ringer’s solution to the desired concentrations^15^. Data acquisition and analysis were carried out with Digidata 1440A and pCLAMP 10.2 software (Axon Instruments Inc., CA). Dose-response data were analyzed by GraphPad Prism 5.0 (GraphPad Software Inc, CA).

### Statistical Analysis

Principle component analysis (PCA) and hierarchical cluster analysis were performed using PASW Statistic 18 (IBM, NY). Euclidean distance and Ward’s method were used for the hierarchical cluster analysis^17^,^35^. PCA was conducted using the correlation matrix.

## Additional Information

**How to cite this article**: Liu, F. *et al*. Molecular Basis of Olfactory Chemoreception in the Common Bed Bug, *Cimex lectularius. Sci. Rep.* **7**, 45531; doi: 10.1038/srep45531 (2017).

**Publisher's note:** Springer Nature remains neutral with regard to jurisdictional claims in published maps and institutional affiliations.

## Supplementary Material

Supplementary Information
