# Supplementary material for: Molecular Basis of Olfactory Chemoreception in the Common Bed Bug, Cimex lectularius
Source: Sci Rep. 2017 Apr 6;7:45531. doi: 10.1038/srep45531 (PMC5382537; doi:10.1038/srep45531)
Supplement: Supplementary Information [file srep45531-s1.pdf]

Molecular Basis of Olfactory Chemoreception in the Common Bed Bug, *Cimex lectularius*

Feng Liu, Zhou Chen, Nannan Liu[§]

Supplement Information

Figure legend

Figure S1. Representative response traces of OR-expressed oocytes to specific compounds. All the compound were tested at the concentration of 10^{-4}. Corresponding control response of raw oocytes to these compounds were showed as the control. The arrow indicated when the compounds were applied in stimulating the oocytes.

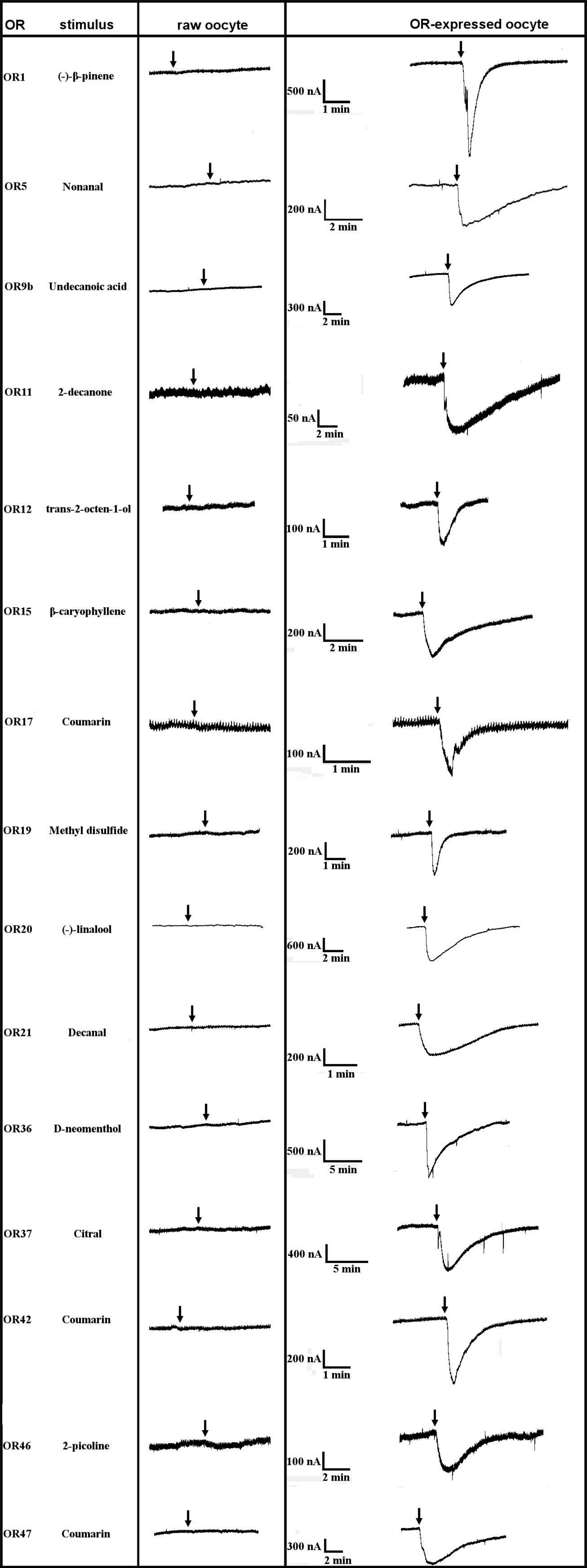

OR
stimulus
raw oocyte
OR-expressed oocyte
OR1
(-)-β-pinene
500 nA
1 min
OR5
Nonanal
200 nA
2 min
OR9b
Undecanoic acid
300 nA
2 min
OR11
2-decanone
50 nA
2 min
OR12
trans-2-octen-1-ol
100 nA
1 min
OR15
β-caryophyllene
200 nA
2 min
OR17
Coumarin
100 nA
1 min
OR19
Methyl disulfide
200 nA
1 min
OR20
(-)-linalool
600 nA
2 min
OR21
Decanal
200 nA
1 min
OR36
D-neomenthol
500 nA
5 min
OR37
Citral
400 nA
5 min
OR42
Coumarin
200 nA
1 min
OR46
2-picoline
100 nA
2 min
OR47
Coumarin
300 nA
2 min

Table S1. Primers for cloning the ORs of bed bugs

Gene[&]	Forword primer*	Reverse primer*
OR1	ccgactagtgccaccATGAGTAAAGTAACGATA	ctaggcggccgcTTAATGTTTCTTCGAATCAG
OR5	ccggctagcgccaccATGTGGAAAGTAGCGAGC	ctaggcggccgcTCACAATTTAGAAGACACCG
OR7	ccggctagcgccaccATGCCAGGGAAAAAGGGT	ctaggcggccgcTTAATTTCCTCTTGAAGCGT
OR8	ccggctagcgccaccATGGCCGGTAAGGAAAAG	ctaggcggccgcTTAATTTTGCGATGCGTTGA
OR9b	ccggctagcgccaccATGGGAACTGTAAAAACA	ctaggcggccgcACCCATGAGGGCTTTGAGTA
OR11	ccggctagcgccaccATGGGTAAAGGAGGATCA	ctaggcggccgcTTATCTCGTGGCTTTTAAGG
OR12b	ccggctagcgccaccATGGCTCAGCTCTTCGAC	ctaggcggccgcTTAATTATCGACAGTTCTTA
OR14	ccggctagcgccaccATGCAATCAAAATGTATC	ctaggcggccgcTCAAATAAATAACAATCGCA
OR15	ccggctagcgccaccATGGTCGGCCGAAGGGAT	ctaggcggccgcTCATTGCAGGCTTTGAAGAA
OR16	ccggctagcgccaccATGAACGAAAATTTAAAA	ctaggcggccgcTCAATACATTGATTTAAGCA
OR17	ccggctagcgccaccATGAACGAGAACATGAAG	ctaggcggccgcTCAATTCATCGATTTGAGTA
OR19	ccggctagcgccaccATGACAGAATTGAAGAAA	ctaggcggccgcTTATATATCGAAATGCTGAA
OR20	ccggctagcgccaccATGAAGTTCGGAAGATAT	ctaggcggccgcTCAAAAGTTCATATTTTTGA
OR21	ccggctagcgccaccATGGCGGTGGATGTAAAG	ctaggcggccgcTCATCTCTCATTTAGGAAAA
OR23	ccggctagcgccaccATGGGAAAAGAGAAAAGT	ctaggcggccgcTTAAGACCTCTCCATTGTCC
OR27	ccggctagcgccaccATGACGGCGCTTTCCGGT	ctaggcggccgcCTATAGTTTTTCGCTTGATG
OR36	ccggctagcgccaccATGGCAAGTATTCAGGAC	ctaggcggccgcTTAAGATTTCGTAGCGATAA
OR37	ccggctagcgccaccATGACCTACTGGAAAGAA	ctaggcggccgcCTATGTCTTGATATCAATCG
OR42	ccggctagcgccaccATGGTTTCGGAAGGGATA	ctaggcggccgcTCAGCGGTTCATTTGTTTAA
OR46	ccggctagcgccaccATGGAGTCGACAGAGATT	ctaggcggccgcTCACTTCTCAAGAACGAACT
OR47	ccggctagcgccaccATGGAAGAGGTGATGTCG	ctaggcggccgcTTAATTGAGTTTCTTCAGCA

&: yellow color showed OR genes expressed in *Xenopus* oocytes with no responses to any odorants used in this experiment. *pink color: protective nucleotides; red color: restriction enzyme cutting site; green color: Kozak sequence; black color: gene specific primer sequence

Table S2. Current responses (Mean value) of bed bug odorant receptors to odorants

Odorants	OR#														
	1	5	9b	11	12	15	17	19	20	21	36	37	42	46	47
myrcene	5	13	5	0	0	30	10	10	315	23	50	47	0	5	10
terpeloene	80	10	0	10	0	15	40	20	240	0	313	0	57	30	20
α-terpinene	40	23	20	0	3	0	5	5	215	88	170	0	0	5	10
α-pinene	245	27	0	0	0	20	20	10	65	20	80	0	5	10	10
(+)-α-pinene	320	43	0	13	0	25	0	20	195	37	410	83	695	3	0
1S-α-(-)-pinene	40	23	10	0	10	0	10	0	20	78	50	17	18	15	0
(+)-β-pinene	640	23	20	3	3	20	5	0	315	60	135	83	35	5	10
(-)-β-pinene	1527	33	25	17	0	10	10	5	55	50	47	0	13	10	0
(1S)-(+)-3-carene	255	27	15	60	17	10	0	15	195	43	105	288	140	5	5
R-(+)-limonene	0	0	5	0	0	15	5	10	225	13	377	0	23	5	5
S-(-)-limonene	35	10	5	17	0	10	50	25	300	57	478	120	20	35	25
β-caryophyllene	0	0	5	7	0	458	215	25	0	30	0	0	15	20	20
(-)-caryophyllene oxide	5	57	5	13	7	55	0	0	0	75	0	0	17	0	20
citral	0	17	35	23	0	35	10	10	235	20	75	840	5	20	10
citronellal	5	0	15	3	0	10	0	0	65	70	0	163	10	0	7
S-(-)-perillaldehyde	25	20	30	50	17	45	30	0	25	23	130	0	30	40	25
citronellol	10	23	20	17	3	0	5	0	250	30	0	0	3	10	0
eugenol	0	0	5	0	0	10	0	45	15	0	0	0	20	30	35
geraniol	0	27	45	13	17	30	20	10	310	27	105	260	0	5	15
menthol	55	40	5	0	3	0	35	30	230	93	690	0	40	45	20
D-neomenthol	75	10	10	0	0	0	0	0	415	0	1552	203	7	173	3
eucalyptol	690	13	25	0	23	10	0	5	23	20	0	33	15	15	10
S-(-)-perillyl alcohol	30	13	30	27	40	50	5	5	50	40	140	0	10	5	10
camphor	540	20	5	7	13	0	20	55	0	20	0	223	40	35	10
thymol	0	10	15	0	0	0	0	5	270	13	60	0	0	5	5
carvacrol	5	3	5	0	0	10	0	0	30	0	0	0	3	0	0
α-terpineol	50	27	15	0	7	5	5	10	605	47	480	0	0	5	10
phytol	40	0	5	3	3	15	5	0	5	17	0	0	3	10	5
(S)-cis-verbenol	760	20	5	0	23	0	25	45	30	33	45	210	175	30	5
(+)-terpinen-4-ol	75	20	35	0	10	15	10	5	393	70	365	0	5	5	15
(-)-linalool	0	7	5	0	33	5	10	5	1230	115	40	0	5	5	10
linalyl acetate	25	13	20	7	67	90	25	15	545	128	0	137	65	10	45
menthyl acetate	5	60	0	7	0	20	25	65	30	27	638	30	15	30	15
geranyl acetate	0	43	20	30	100	10	20	50	160	100	0	440	45	25	40
geranyl acetone	15	3	10	60	43	0	0	5	135	80	27	0	0	5	0
(+)-menthone	115	10	0	27	7	30	3	63	265	17	250	185	55	243	17
(-)-menthone	45	0	5	20	10	5	5	0	315	17	768	130	33	25	15
(-)-α-tunjone	370	3	5	0	0	40	30	25	85	0	0	108	30	35	25
citronellic acid	70	43	55	30	23	65	5	0	75	0	27	63	47	5	15
1-chlorohexane	20	30	20	3	0	27	17	23	0	40	0	10	27	30	23
1-chloroheptane	15	50	15	17	10	17	10	40	0	23	13	17	3	27	33
1-chlorohexadecane	25	47	10	30	10	0	23	23	40	17	0	10	23	13	27
1-chlorododecane	10	50	15	37	0	17	3	13	5	23	0	3	3	40	30
1-chlorotetradecane	5	23	10	13	20	20	13	17	65	17	0	0	8	0	10

benzyl chloride	0	57	0	0	0	0	63	0	0	0	67	0	0	0	0
ammonia	0	0	0	0	0	0	0	0	0	0	0	0	0	0	0
butylamine	0	40	0	0	17	0	0	0	0	0	0	0	0	0	7
propylamine	0	20	0	0	0	35	0	0	30	0	0	0	0	0	0
1-tetradecene	10	23	20	7	3	20	0	20	25	33	10	10	5	0	0
1-hexadecene	10	43	15	0	0	5	13	27	5	37	0	0	20	30	7
2,4-dimethyl hexane	0	57	0	13	0	15	0	3	0	20	0	0	17	7	10
2-pentene	15	47	5	3	0	15	0	20	30	17	0	0	20	0	10
hexane	0	20	20	3	0	25	0	50	0	40	15	0	0	40	40
heptane	0	7	0	7	0	0	0	27	0	0	0	0	20	10	23
octane	0	7	15	7	0	0	0	23	0	17	0	0	15	0	0
trans-2-octene	25	13	25	13	0	20	0	30	75	23	128	3	20	13	10
trans-3-octene	40	0	10	3	3	15	7	45	20	7	1063	7	15	13	17
trans-4-octene	15	13	10	13	0	23	10	10	15	17	768	3	20	20	17
nonane	0	13	15	0	0	5	0	17	0	7	0	0	17	3	3
decane	0	10	10	0	0	0	0	17	0	17	0	0	13	17	0
pentadecane	5	30	20	3	3	0	0	0	10	13	0	0	20	0	7
heptadecane	0	13	5	7	0	10	0	3	5	23	0	0	10	0	3
benzene	493	10	15	0	23	25	0	130	35	20	0	20	23	23	273
ethyl benzene	130	20	10	10	3	10	25	60	160	43	78	0	15	10	20
propylbenzene	290	23	20	13	0	5	20	35	175	30	43	10	10	10	5
styrene	490	40	10	13	7	35	40	30	610	57	60	40	15	50	25
toluene	245	13	10	3	13	0	35	65	70	33	78	0	40	15	15
xylene	630	17	5	3	0	10	15	55	35	30	275	0	5	15	15
squalene	0	3	5	10	0	20	0	23	5	10	0	0	17	7	3
cinnamyl alcohol	30	10	35	10	0	35	5	10	230	13	27	0	53	0	10
isoamyl alcohol	50	33	10	10	17	25	5	0	105	27	25	0	0	10	5
1-hexen-3-ol	225	13	30	7	13	0	17	85	765	87	875	77	5	23	37
1-octen-3-ol	115	30	10	17	0	70	67	38	650	133	983	30	30	50	30
cis-2-hexen-1-ol	265	30	25	17	13	55	10	50	35	40	90	13	10	20	10
trans-2-hexen-1-ol	110	23	25	7	17	35	15	78	295	33	85	13	10	15	20
trans-2-octen-1-ol	85	0	10	10	150	15	10	70	263	77	50	0	10	15	10
2-decanol	0	30	0	0	7	10	7	27	20	103	0	0	8	23	33
2-hexadecanol	5	23	10	93	0	20	0	27	5	10	0	0	23	0	13
glycerol	15	30	5	7	10	20	10	27	50	13	0	0	30	13	0
phenethyl alcohol	225	33	5	33	40	65	0	123	205	50	70	30	35	15	5
o-cresol	260	7	20	0	3	25	7	3	35	20	20	0	0	3	213
phenol	20	0	25	0	0	20	0	63	125	3	0	0	27	10	127
4-ethylphenol	90	17	5	7	20	20	0	207	45	57	25	0	20	5	5
coumarin	750	13	5	3	47	13	423	17	60	3	27	43	640	213	580
pyrazine	40	10	15	10	0	5	0	30	10	3	20	7	20	5	5
indole	325	17	0	10	0	23	0	60	0	10	0	0	15	5	15
skatole	153	0	0	10	0	13	0	0	35	0	5	0	28	0	603
2-picoline	240	13	0	10	20	0	0	210	175	17	0	17	578	363	447
3-aminopyridine	5	13	15	7	10	5	0	23	5	13	0	0	35	33	67
1-methyl piperidine	0	0	0	0	0	0	0	0	0	0	0	0	0	0	0
1-piperidineethanol	0	0	0	0	0	0	0	0	0	0	0	0	0	0	0
thiazolidine	15	13	15	3	0	40	0	27	60	0	45	0	50	0	5

2-methylfunan	45	30	30	20	7	40	23	37	10	60	15	47	210	30	183
2,6-dimethylpyrazine	220	17	10	0	0	17	5	103	0	10	65	13	320	180	10
4-piperidinemethanamine	0	0	0	0	0	0	0	0	0	0	0	0	0	0	0
2-butanone	30	37	10	13	0	5	20	248	100	27	30	50	70	15	20
2-penanone	140	43	5	7	0	0	10	311	150	27	183	67	150	10	10
3-pentanone	65	13	25	0	7	20	20	203	220	27	337	138	185	25	15
2-hexanone	237	17	5	15	20	40	30	197	495	27	833	220	135	35	30
2-decanone	30	27	20	197	27	13	35	27	57	53	650	20	10	30	25
sulcatone	413	23	20	20	120	70	35	95	547	17	140	70	20	30	35
propional	35	100	35	0	0	110	60	236	10	97	187	97	80	40	65
butanal	35	43	10	60	0	50	45	268	110	108	473	113	65	45	85
isobutanal	50	60	45	30	0	80	35	57	25	63	177	63	60	30	30
2-methylbutanal	168	30	0	28	0	87	20	180	20	40	155	103	135	35	15
pentanal	135	40	45	43	0	83	50	246	65	57	153	87	65	35	45
hexanal	175	60	40	60	0	97	55	157	210	73	173	53	40	45	50
heptanal	245	33	65	13	10	117	50	120	185	33	120	27	60	45	45
octanal	150	117	25	75	90	83	5	100	150	162	593	33	70	10	0
nonanal	10	277	50	20	27	30	60	24	70	294	310	20	20	50	40
decanal	25	43	120	25	13	23	25	28	47	458	120	10	20	20	20
benzaldehyde	1337	50	10	0	73	27	45	50	177	90	43	150	430	45	121
Tran-cinnamalldehyde	143	40	50	0	0	40	63	83	130	30	80	0	77	30	53
acetic acid	0	0	0	0	0	0	0	0	0	0	0	0	0	0	0
propiolic acid	0	0	0	0	0	0	0	0	0	0	0	0	0	57	0
butylic acid	0	0	0	0	0	0	0	0	35	0	0	0	0	0	0
valeric acid	0	0	0	0	3	0	0	0	35	0	0	0	0	0	0
hexanoic acid	0	0	0	0	0	0	57	0	50	0	0	0	0	0	53
heptanoic acid	20	0	10	23	7	20	3	43	30	0	40	13	25	30	17
octanoic acid	65	57	60	0	0	60	33	83	45	0	0	0	55	53	0
nonanoic acid	60	57	0	0	0	0	57	0	60	0	0	50	0	53	0
decanoic acid	70	0	335	0	0	65	33	87	0	0	0	0	0	53	53
undecanoic acid	0	50	525	0	47	60	37	47	0	0	0	47	55	47	63
dodecanoic acid	40	27	450	37	7	25	0	37	15	0	40	27	40	37	33
tridecanoic acid	75	53	175	30	0	30	30	0	45	0	60	57	45	37	50
pentadecanoic acid	35	20	35	20	3	55	20	7	0	27	20	7	30	17	23
oleic acid	10	0	15	0	0	5	5	0	0	30	0	0	20	0	5
linoleic acid	30	30	35	33	27	30	30	30	0	43	40	13	20	40	35
acrylic acid	0	0	0	0	0	0	0	0	0	0	0	0	0	0	0
adipic acid	0	0	0	0	0	0	0	0	0	0	0	0	0	0	0
pimelic acid	0	0	0	0	0	0	0	0	0	0	0	0	0	0	0
myristic acid	35	7	50	10	0	10	27	63	55	0	20	53	37	30	23
L-(+)-lactic acid	0	0	0	0	0	0	0	0	0	0	0	0	0	0	0
benzoic acid	0	0	0	0	0	0	0	0	0	0	0	0	0	0	0
4-hydrobenzoic acid	40	43	50	0	0	0	40	0	40	0	90	0	0	0	60
DL-3-methylvaleric acid	0	57	0	0	0	0	0	0	0	0	0	0	0	0	0
trans-2,3-dimethylacrylic acid	0	0	0	0	0	0	20	0	0	0	0	0	0	0	15
methyl nanonate	55	180	45	50	37	40	0	20	30	63	275	0	10	0	0

methyl tridecanoate	20	0	20	33	20	5	5	17	20	43	10	10	10	0	0
dibutyl phthalate	0	3	15	0	3	40	0	0	5	35	0	0	7	0	0
dimethyl phthalate	5	10	5	0	0	40	0	0	120	23	688	0	33	5	50
carbon disulfide	320	17	20	10	13	30	10	20	35	47	20	17	20	13	13
methyl disulfide	360	40	25	3	43	40	7	457	90	50	53	17	185	13	150
urea	0	0	0	0	0	10	0	13	0	10	0	0	10	0	0
thiourea	0	0	0	0	0	0	0	3	10	0	0	0	5	0	0
methyl urea	15	0	0	0	0	0	0	20	0	20	0	0	20	0	0
menthoglycol	5	10	5	0	3	10	0	5	13	17	53	47	0	0	0
naphthalene	1170	37	0	3	3	10	70	45	10	93	0	7	43	20	30

Table S3. SEM of current responses of bed bug odorant receptors to odorants

Odorants	OR#														
	**1**	**5**	**9b**	**11**	**12**	**15**	**17**	**19**	**20**	**21**	**36**	**37**	**42**	**46**	**47**
myrcene	5	3	5	0	0	4	0	0	5	15	10	12	0	5	0
terpeloene	10	0	0	0	0	2	0	0	38	0	41	0	3	10	0
α-terpinene	10	15	0	0	3	0	5	4	35	5	10	0	0	5	0
α-pinene	45	12	0	0	0	0	0	0	5	6	20	0	5	0	0
(+)-α-pinene	55	9	0	3	0	2	0	0	15	7	21	9	35	3	0
1S-α-(-)-pinene	10	12	0	0	10	0	10	0	10	19	6	3	5	5	0
(+)-β-pinene	120	7	0	3	3	0	5	0	45	10	35	7	5	5	0
(-)-β-pinene	376	3	15	3	0	0	0	4	15	25	9	0	3	0	0
(1S)-(+)-3-carene	5	12	5	40	3	0	0	11	15	7	5	43	0	5	5
R-(+)-limonene	0	0	5	0	0	2	5	7	5	3	87	0	3	5	5
S-(-)-limonene	5	0	5	3	0	0	0	11	60	18	67	20	10	15	15
β-caryophyllene	0	0	5	3	0	23	25	4	0	0	0	0	5	10	0
(-)-caryophyllene oxide	5	9	5	3	7	2	0	0	0	3	0	0	3	0	10
citral	0	3	5	3	0	2	0	0	35	12	5	49	5	0	0
citronellal	5	0	5	3	0	0	0	0	25	26	0	25	0	0	3
S-(-)-perillaldehyde	25	6	0	0	7	2	20	0	5	3	30	0	0	10	5
citronellol	10	7	10	3	3	0	5	0	60	6	0	0	3	10	0
eugenol	0	0	5	0	0	0	0	11	5	0	0	0	10	0	25
geraniol	0	9	5	3	3	0	0	0	50	12	5	12	0	5	5
menthol	5	6	5	0	3	0	15	7	50	11	193	0	10	5	10
D-neomenthol	35	6	0	0	0	0	0	0	15	0	151	20	3	46	3
eucalyptol	70	9	5	0	15	4	0	4	3	6	0	13	5	15	0
S-(-)-perillyl alcohol	10	3	10	7	6	0	5	4	10	0	10	0	0	5	10
camphor	60	6	5	3	3	0	0	11	0	6	0	19	20	5	0
thymol	0	6	5	0	0	0	0	4	46	3	0	0	0	5	5
carvacrol	5	3	5	0	0	0	0	0	10	0	0	0	3	0	9
α-terpineol	20	12	5	0	7	2	5	0	105	13	50	0	0	5	0
phytol	10	0	5	3	3	2	5	0	5	9	0	0	3	10	5
(S)-cis-verbenol	120	15	5	0	15	0	15	11	10	3	25	17	75	10	5
(+)-terpinen-4-ol	15	6	5	0	0	2	0	4	87	10	35	0	5	5	5
(-)-linalool	0	3	5	0	24	2	0	4	97	29	20	0	5	5	0
linalyl acetate	5	12	0	7	12	0	15	4	25	9	0	68	35	0	5
menthyl acetate	5	20	0	3	0	0	5	11	10	3	244	12	5	0	5
geranyl acetate	0	12	10	0	6	0	0	28	10	12	0	95	5	5	0
geranyl acetone	5	3	0	12	9	0	0	4	25	18	3	0	0	5	0
(+)-menthone	5	0	0	7	7	0	3	10	15	3	34	31	5	39	7
(-)-menthone	5	0	5	0	0	2	5	0	45	3	183	21	3	15	5
(-)-α-tunjone	40	3	5	0	0	7	10	4	15	0	0	14	10	15	5
citronellic acid	10	13	5	0	3	2	5	0	15	0	3	7	3	5	15
1-chlorohexane	0	6	0	3	0	1	3	3	0	6	0	6	17	6	3
1-chloroheptane	5	10	5	12	10	3	6	15	0	3	6	17	3	3	7
1-chlorohexadecane	5	17	0	0	0	0	3	3	0	3	0	6	3	3	7
1-chlorododecane	0	15	5	3	0	1	3	3	5	3	0	3	3	6	6
1-chlorotetradecane	5	3	0	7	6	0	3	3	5	3	0	0	3	0	6

benzyl chloride	0	3	0	0	0	0	3	0	0	0	3	0	9	0	0
ammonia	0	0	0	0	0	0	0	0	0	0	0	0	0	0	0
butylamine	0	21	0	0	3	0	0	0	0	0	0	0	0	0	3
propylamine	0	6	0	0	0	2	0	0	0	0	0	0	0	0	0
1-tetradecene	0	3	0	3	3	0	0	7	5	3	4	0	5	0	0
1-hexadecene	10	9	5	0	0	2	9	3	5	3	0	0	0	6	7
2,4-dimethyl hexane	0	3	0	7	0	2	0	3	0	6	0	0	3	3	0
2-pentene	5	7	5	3	0	2	0	0	10	3	0	0	0	0	6
hexane	0	10	10	3	0	2	0	16	0	10	5	0	0	0	6
heptane	0	3	0	3	0	0	0	3	0	0	0	0	0	0	13
octane	0	3	5	3	0	0	0	18	0	7	0	0	5	0	0
trans-2-octene	5	3	5	7	0	4	0	4	25	3	12	3	0	3	6
trans-3-octene	10	0	0	3	3	2	3	4	10	3	221	3	15	3	3
trans-4-octene	5	3	0	7	0	1	0	4	5	3	159	3	12	0	3
nonane	0	3	5	0	0	2	0	3	0	3	0	0	3	3	3
decane	0	6	0	0	0	0	0	3	0	3	0	0	7	3	0
pentadecane	5	0	10	3	3	0	0	0	10	3	0	0	0	6	3
heptadecane	0	3	5	3	0	0	0	3	5	3	0	0	0	0	3
benzene	121	6	5	0	3	2	0	12	15	0	0	0	3	3	17
ethyl benzene	20	6	0	0	3	0	5	0	0	15	17	0	5	0	20
propylbenzene	30	3	0	3	0	2	10	4	15	15	3	0	10	10	5
styrene	180	6	0	3	3	2	20	0	10	3	0	0	5	0	5
toluene	75	3	0	3	3	0	15	4	20	3	3	0	30	5	5
xylene	90	3	5	3	0	4	5	4	5	6	76	0	5	5	5
squalene	0	3	5	6	0	0	0	3	5	6	0	0	3	3	3
cinnamyl alcohol	0	6	5	0	0	2	5	7	10	3	3	0	3	0	10
isoamyl alcohol	20	12	10	0	7	5	5	0	35	3	5	0	0	10	5
1-hexen-3-ol	25	3	0	3	3	0	3	18	85	9	26	3	5	7	12
1-octen-3-ol	25	0	0	3	0	4	13	6	10	19	80	0	0	6	6
cis-2-hexen-1-ol	5	6	5	3	3	2	0	7	5	10	0	7	10	10	0
trans-2-hexen-1-ol	20	3	5	3	3	2	5	20	35	9	25	7	10	5	0
trans-2-octen-1-ol	15	0	0	6	26	2	0	19	72	9	9	0	0	5	0
2-decanol	0	0	0	0	3	0	3	3	10	16	0	0	3	3	9
2-hexadecanol	5	3	0	6	0	0	0	3	5	6	0	0	3	0	3
glycerol	5	6	5	3	0	0	0	5	10	3	0	0	6	3	0
phenethyl alcohol	25	3	5	3	6	0	0	57	15	10	20	0	5	5	5
o-cresol	80	3	0	0	3	2	3	3	5	0	10	0	0	3	19
phenol	10	0	5	0	0	4	0	3	25	3	0	0	3	0	33
4-ethylphenol	20	3	5	3	6	0	0	90	15	3	5	0	0	5	5
coumarin	110	3	5	3	7	2	92	3	30	3	7	7	116	30	133
pyrazine	20	6	5	0	0	2	0	15	0	3	0	3	10	5	5
indole	145	3	0	6	0	3	0	25	0	6	0	0	5	5	5
skatole	32	0	0	0	0	1	0	0	5	0	5	0	5	0	99
2-picoline	130	7	0	6	0	0	0	12	25	3	0	9	109	47	64
3-aminopyridine	5	9	5	3	0	2	0	3	5	7	0	0	9	3	15
1-methyl piperidine	0	0	0	0	0	0	0	0	0	0	0	0	0	0	0
1-piperidineethanol	0	0	0	0	0	0	0	0	0	0	0	0	0	0	0
thiazolidine	5	13	5	3	0	0	0	17	10	0	5	0	10	0	5

2-methylfunan	15	6	10	0	3	0	3	5	0	6	5	3	25	0	48
2,6-dimethylpyrazine	52	7	0	0	0	1	5	76	0	6	15	7	50	10	0
4-piperidinemethanamine	0	0	0	0	0	0	0	0	0	0	0	0	0	0	0
2-butanone	10	7	0	3	0	2	0	39	51	9	6	12	20	5	0
2-penanone	40	3	5	3	0	0	0	53	10	3	48	34	0	0	0
3-pentanone	5	7	5	0	3	0	0	36	47	3	92	8	15	5	5
2-hexanone	66	3	5	3	12	0	0	38	95	3	186	34	5	5	0
2-decanone	10	9	10	29	12	1	15	2	22	7	132	6	0	10	5
sulcatone	139	3	0	7	11	4	5	24	134	7	60	6	0	0	5
propional	5	0	5	0	0	2	20	30	0	22	7	7	0	0	5
butanal	15	3	0	6	0	4	15	38	10	15	50	5	15	5	15
isobutanal	20	6	5	7	0	2	5	5	5	3	7	3	20	0	10
2-methylbutanal	37	6	0	3	0	1	0	58	0	6	35	14	15	15	5
pentanal	15	6	5	5	0	2	10	29	5	19	9	3	15	5	5
hexanal	25	6	10	13	0	3	15	47	20	3	7	3	0	5	10
heptanal	55	3	5	3	0	1	0	21	15	3	6	3	10	5	5
octanal	20	12	5	14	47	1	5	15	0	29	41	7	10	0	0
nonanal	10	58	0	4	3	0	0	3	0	17	6	0	0	0	0
decanal	5	12	10	3	3	1	5	3	12	65	0	0	10	0	0
benzaldehyde	363	6	10	0	9	1	5	8	44	15	15	24	10	5	5
trans cinnamalldehyde	35	6	0	0	0	0	17	10	0	0	6	0	3	10	13
acetic acid	0	0	0	0	0	0	0	0	0	0	0	0	0	0	0
propiolic acid	0	0	0	0	0	0	0	0	0	0	0	0	0	30	0
butylic acid	0	0	0	0	0	0	0	0	15	0	0	0	0	0	0
valeric acid	0	0	0	0	3	0	0	0	5	0	0	0	0	0	0
hexanoic acid	0	0	0	0	0	0	15	0	10	0	0	0	0	0	3
heptanoic acid	0	0	10	7	7	0	3	7	10	0	0	3	5	0	3
octanoic acid	5	9	10	0	0	4	9	7	5	0	0	0	5	7	0
nonanoic acid	0	9	0	0	0	0	7	0	0	0	0	0	0	7	0
decanoic acid	20	0	75	0	0	2	3	7	0	0	0	0	0	7	9
undecanoic acid	0	6	0	15	3	0	3	3	0	0	0	3	5	3	9
dodecanoic acid	10	7	30	3	3	2	0	3	5	7	0	13	10	3	3
tridecanoic acid	15	9	45	0	0	0	6	0	5	0	10	3	5	3	6
pentadecanoic acid	5	6	5	0	3	2	6	3	0	7	0	7	10	3	3
oleic acid	0	0	5	0	0	2	5	0	0	6	6	0	4	0	5
linoleic acid	0	3	5	9	3	0	0	0	20	9	10	3	0	0	5
acrylic acid	0	0	0	0	0	0	0	0	0	0	0	0	0	0	0
adipic acid	0	0	0	0	0	0	0	0	0	0	0	0	0	0	0
pimelic acid	0	0	0	0	0	0	0	0	0	0	0	0	0	0	0
myristic acid	15	3	0	0	0	0	3	3	15	0	10	3	3	6	3
L-(+)-lactic acid	0	0	0	0	0	0	0	0	0	0	0	0	0	0	0
benzoic acid	0	0	0	0	0	0	0	0	0	0	0	0	0	0	0
4-hydrobenzoic acid	0	3	0	0	0	0	0	0	10	0	0	0	0	0	10
DL-3-methylvaleric acid	0	3	0	0	0	0	0	5	0	0	0	0	0	0	0
trans-2,3-dimethylacrylic acid	0	0	0	0	0	0	0	0	0	0	0	0	0	0	5
methyl nanonate	15	31	5	0	3	0	0	4	0	3	25	0	0	0	0
methyl tridecanoate	0	0	10	3	0	2	5	3	10	9	10	0	0	0	0

dibutyl phthalate	0	3	5	0	3	0	0	0	5	5	0	0	3	0	0
dimethyl phthalate	5	6	5	0	0	4	0	0	10	3	41	0	9	5	50
carbon disulfide	100	3	10	6	3	0	6	0	5	15	0	3	0	3	3
methyl disulfide	40	12	5	3	9	0	3	123	20	32	18	9	75	3	21
urea	0	0	0	0	0	4	0	3	0	6	0	0	10	0	0
thiourea	0	0	0	12	0	0	0	3	0	0	0	0	5	0	0
methyl urea	15	0	0	0	0	0	0	8	0	6	0	0	0	0	0
menthoglycol	5	0	5	0	3	4	0	4	3	3	9	18	0	0	0
naphthalene	130	15	0	3	3	4	10	11	0	9	0	3	3	0	30
